# Successful treatment with hyperbaric oxygen therapy for ulcerative esophageal stricture after chemoradiotherapy: A case report

**DOI:** 10.1002/deo2.70072

**Published:** 2025-01-29

**Authors:** Tetsuyuki Tateda, Hidezumi Kikuchi, Keisuke Furusawa, Yusuke Matsuoka, Taka Asari, Yohei Sawada, Satoru Nakagawa, Tatsuta Tetsuya, Daisuke Chinda, Hirotake Sakuraba

**Affiliations:** ^1^ Department of Gastroenterology, Hematology, and Clinical Immunology Hirosaki University Graduate School of Medicine Aomori Japan; ^2^ Department of Gastroenterology, Hematology, and Clinical Immunology Odate Municipal General Hospital Akita Japan; ^3^ Department of Community Medicine Hirosaki University Graduate School of Medicine Aomori Japan; ^4^ Division of Endoscopy Hirosaki University Hospital Aomori Japan

**Keywords:** chemoradiotherapy, digestive system endoscopy, dilation, esophageal stenosis, hyperbaric oxygenation

## Abstract

Severe esophageal strictures resulting from chemoradiotherapy pose persistent therapeutic challenges despite the availability of treatments such as endoscopic balloon dilation and medications. Hyperbaric oxygen therapy (HBOT) has emerged as a promising treatment option for refractory radiation‐induced injury to several organs. Herein, we present the case of a 79‐year‐old male patient with refractory radiation‐induced ulcerative esophageal strictures after chemoradiotherapy. Despite multiple interventions, including endoscopic balloon dilation, steroids, and proton‐pump inhibitors, the patient remained unable to tolerate oral intake. HBOT was initiated, leading to significant improvement in the esophageal ulcers and strictures within 1 month. HBOT was well tolerated; the patient experienced a sustained improvement in his quality of life. Two years after HBOT, esophagogastroduodenoscopy confirmed persistent improvement in esophageal ulcers and strictures. This case highlights the potential of HBOT as a therapeutic option for ulcerative esophageal strictures unresponsive to conventional treatments.

## INTRODUCTION

Esophageal strictures have various causes, including congenital narrowing, achalasia, esophageal cancer, surgical anastomoses, ulcers, and gastroesophageal reflux disease. Particularly, refractory ulcerative esophageal strictures following chemoradiotherapy (CRT) pose ongoing therapeutic challenges. Although common treatments, such as nutritional support, medication, or endoscopic balloon dilation (EBD) are available and widely performed for ulcerative esophageal stricture,^1^, ^2^, ^3^ they proved ineffective in some patients. Treatment‐resistant strictures impair oral intake and substantially diminish patient's quality of life.

Hyperbaric oxygen therapy (HBOT) is a minimally invasive treatment with few adverse effects. It has shown efficacy in managing various conditions including radiation‐induced proctitis.^4^, ^5^ In this report, we present a case of successful resolution of radiation‐induced esophageal stricture following HBOT. In our case, ulcerative esophageal stricture after CRT that was resistant to conventional multiple treatments was epithelized without stenosis by HBOT. This case underscores the potential of HBOT as a therapeutic option in patients with ulcerative esophageal strictures resistant to conventional treatments.

## CASE REPORT

**FIGURE 1:**
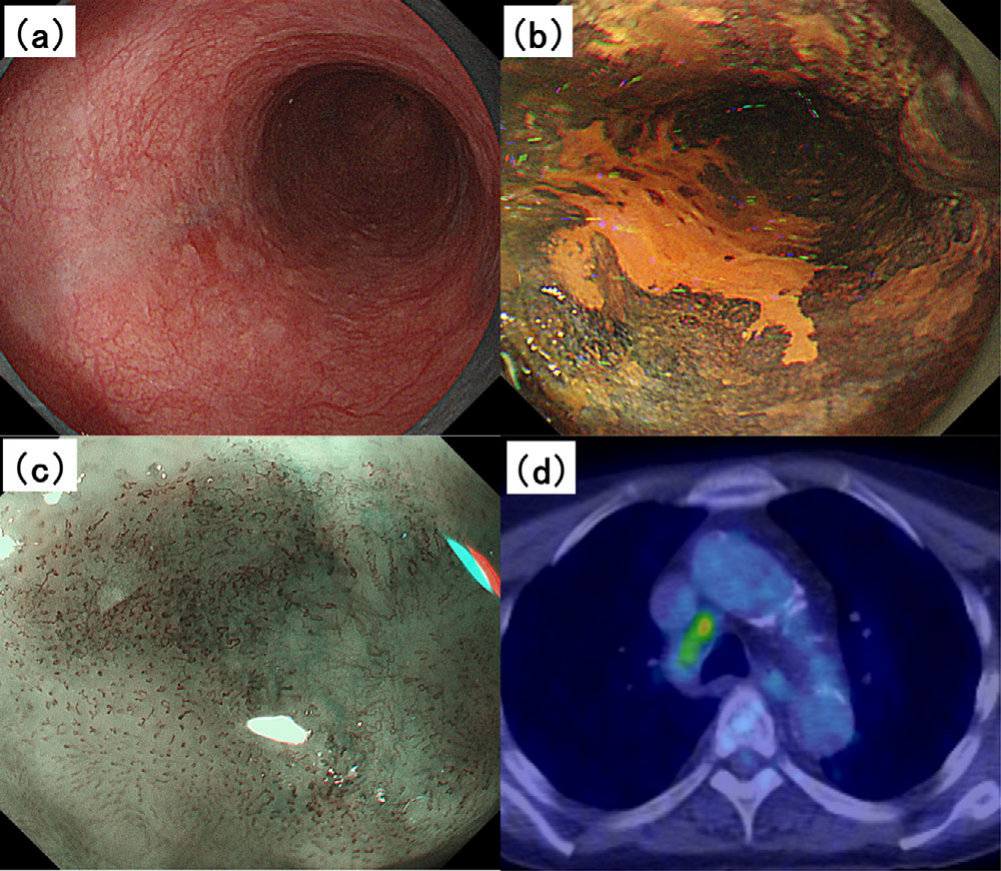
(a) EGD revealed a flat lesion in the esophagus, covering approximately half of its circumference. (b) Chromoendoscopy showed non‐staining with iodine. (c) Magnifying endoscopy with narrow‐band imaging revealed severe irregular vessels including type B2. (d) PET‐CT scan detected metastasis to the peritracheal lymph nodes. EGD, esophagogastroduodenoscopy; HBOT, hyperbaric oxygen therapy; PET‐CT, positron emission tomography‐computed tomography.

A 79‐year‐old man, an ex‐smoker with a history of consuming two liters of beer daily, presented to our hospital. Esophagogastroduodenoscopy (EGD) revealed a 20‐mm flat lesion occupying approximately half of the circumference of the midthoracic esophagus, wherein chromoendoscopy revealed no staining with iodine. Magnifying endoscopy with narrow‐band imaging revealed severe irregular vessels including type B2, according to the classification of the Japan Esophageal Society, with a predicted invasion depth of T1a‐MM or T1b‐SM1 (Figure 1a–c).^6^ Histopathological examination confirmed squamous cell carcinoma. Positron emission tomography‐computed tomography revealed esophageal cancer with metastasis to the peritracheal lymph nodes (Figure 1d). Our cancer board recommended proceeding with CRT considering the patient's advanced age.

**FIGURE 2:**
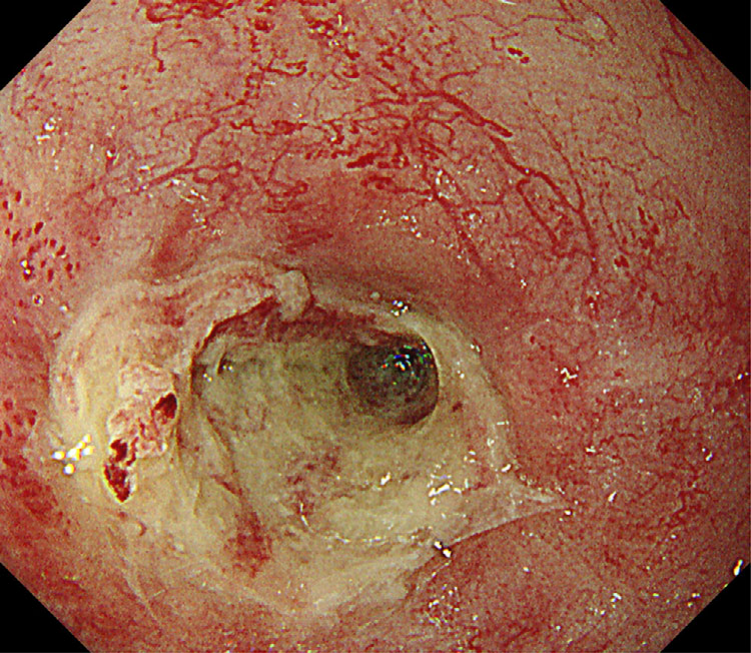
At 3 months post‐initial discharge, EGD revealed ulcers and strictures in the midthoracic esophagus. EGD, esophagogastroduodenoscopy.

During initial hospitalization, the patient underwent CRT for esophageal cancer, comprising systemic chemotherapy (cisplatin+5‐fluorouracil, two cycles) and radiotherapy (60 Gy/30 fractions). Grade 3 leukopenia/neutropenia was observed during treatment but improved upon discharge. Three months after discharge, the patient was readmitted due to dysphagia and inability to tolerate oral intake. EGD revealed a circumferential active ulcer with strictures in the midthoracic esophagus (Figure 2). Even with a small‐caliber endoscope (GIF‐1200N; Olympus), passage through the ulcerated section of the esophagus was impeded, demonstrating a severe stricture. During the second hospitalization, the patient underwent EBD (up to 8 mm in diameter) in addition to intravenous medications, prednisolone (PSL; 30 mg, equivalent to 0.5 mg/kg), and lansoprazole (LPZ, 30 mg). However, EGD, 2 weeks after EBD, showed no change in the esophageal ulcer. Therefore, repeated EBD was not conducted due to the risk of perforation. With continuous PSL and LPZ administration, the patient could intake a liquid diet without dysphagia or chest pain. The patient was discharged on oral medications, PSL, and LPZ, requesting discharge to live at home.

**FIGURE 3:**
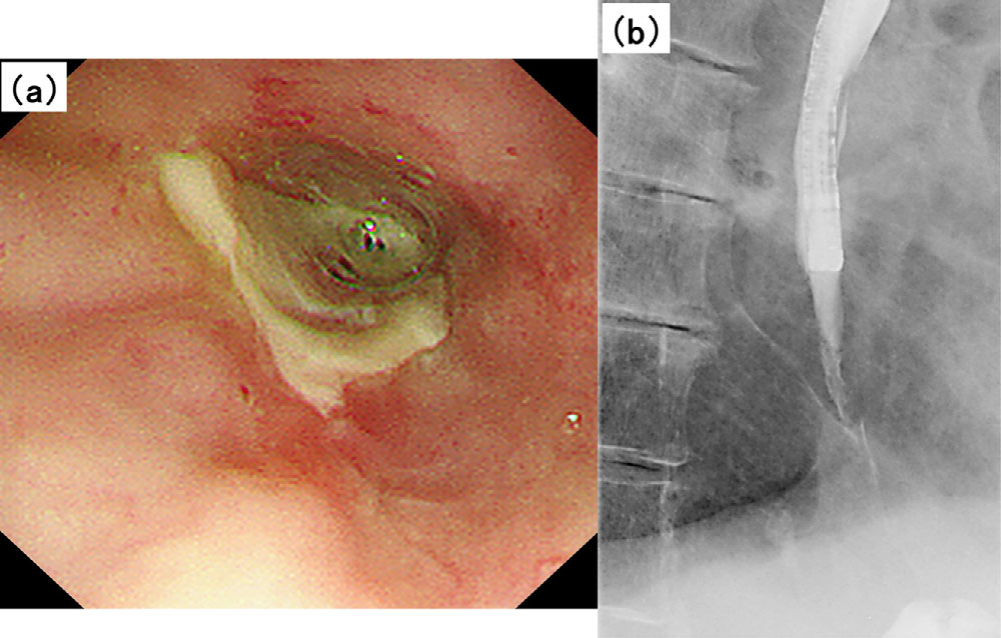
(a, b) At the time of the third admission, EGD and radiography examination showed no improvement in esophageal ulcers and strictures, impeding passage of the endoscope. EGD, esophagogastroduodenoscopy.

**FIGURE 4:**
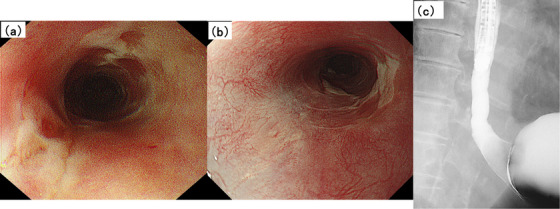
(a) After 10 HBOT sessions, EGD revealed improvement in esophageal ulcers and strictures, allowing passage of the endoscope. (b, c) EGD and radiography conducted 3 months after completing 20 HBOT sessions demonstrated sustained improvement in esophageal ulcers and strictures. EGD, esophagogastroduodenoscopy; HBOT, hyperbaric oxygen therapy.

Two months after the second discharge, the liquid diet caused chest pain. Therefore, the patient was urgently admitted to the hospital for a third time before performing a follow‐up EGD. After hospitalization, EGD revealed no improvement in the esophageal circumferential ulcer with stricture (Figure 3). Despite intravenous re‐administrations of PSL (30 mg) and LPZ (30 mg), the patient remained unable to swallow a liquid diet, indicating drug refractory. Based on the patient's history, HBOT was chosen as the next treatment for esophageal ulcers with severe stricture. HBOT for radiation‐induced esophageal injury has not been considered common because conventional treatments show efficacy, to some extent. In Japan, although insurance coverage for HBOT includes “peripheral circulatory disorders accompanied by intractable ulcers” and “radiation damage”, there are no organ limitations. After explaining to the patient that HBOT was not a common treatment for radiation‐induced esophageal injury, we obtained his consent. Japanese insurance covers up to 30 HBOT sessions for the treatment of chronic conditions. However, in our setting, a total of 20 sessions (up to 2 atm, 5 sessions/week, for 4 weeks), was based on the advice from our intensivist that 20 HBOT sessions were sufficient to treat the late effects of radiation mucosal injury. After 10 HBOT sessions, the patient tolerated oral intake. EGD showed epithelialization of the esophageal ulcers without strictures, allowing passage of the endoscope (Figure 4a). Japanese insurance recommends receiving 10 HBOT sessions to treat acute conditions such as carbon monoxide poisoning; so in this case, 20 HBOT sessions were conducted as scheduled to treat chronic conditions. Since the patient's condition improved steadily, EBD was not conducted during HBOT treatment. The symptoms of dysphagia and esophageal pain resolved; the patient was able to resume a normal diet. Nutritional counseling was provided after 20 HBOT sessions; the patient was discharged.

Three months, 1 year, and 2 years after HBOT completion, EGD confirmed sustained improvement in esophageal ulcers and strictures. Esophageal radiography also revealed an improvement in peristaltic movement, without strictures (Figure 4b,c). In addition, histopathological examination of esophageal biopsies revealed mild fibrosis without evidence of active inflammation.

## DISCUSSION

Herein, we describe that HBOT is an effective therapy for ulcerative esophageal strictures after CRT. Previously, a few reports have described the efficacy of HBOT in radiation‐induced esophageal injury.^7^, ^8^ Our report emphasizes that HBOT was effective as a non‐stricture‐resulting treatment of ulcerative esophageal strictures after CRT, even in cases resistant to conventional multiple treatments. The patient underwent successful HBOT without any observed adverse effects and was able to resume oral intake, leading to a marked improvement in quality of life.

Regarding the conventional treatment of esophageal ulcers and strictures, pharmacological therapies such as proton‐pump inhibitors (PPIs), mucosal protective agents, and steroids are used.^2^ However, they may not always provide adequate improvement. Similarly, a guideline for radiation esophagitis recommends that esophageal injury be treated with medication or nutritional support.^1^ Further treatment options include EBD for esophageal strictures. However, these procedures carry the risk of bleeding or perforation.^3^ Consequently, effective treatment of esophageal strictures with ulcers is challenging. In this case, despite multiple attempts, EBD, PPI, and PSL resulted in inadequate relief of the obstruction and recurrent strictures. Esophageal strictures significantly reduce patient's quality of life. Therefore, HBOT was selected as an additional minimally invasive treatment.

In previous reports, HBOT enhances fibroblast proliferation, neovascularization, bactericidal effects of white blood cells, epithelialization, and collagen synthesis. It also promotes the healing process of damaged tissues by increasing the oxygen concentration in necrotic tissues.^4^, ^8^ Therefore, HBOT has been effective in treating ulcers caused by peripheral circulation disorders and used to treat necrotic tissues after radiotherapy,^4^, ^5^, ^7^, ^8^, ^9^ suggesting a role in healing esophageal mucosa. Hence, HBOT is a promising option for the treatment of radiation‐induced ulcerative esophageal strictures.

In this case, the remarkable therapeutic outcomes of HBOT included the healing of esophageal ulcers without strictures. Typically, when extensive gastrointestinal ulcers improve, subsequent strictures impede dietary intake. A noteworthy aspect of this case is that both the esophageal ulcer and constriction healed with HBOT. Two years after HBOT, no recurrence of esophageal constriction was observed on EGD.

HBOT is associated with a low incidence of adverse events. Reported adverse effects of HBOT include nausea, headache, subcutaneous emphysema, ear pain, and tinnitus, without reports of fatal outcomes.^4^, ^9^ In cancer patients, one signature benefit of HBOT lies in its safety profile, since these patients are particularly susceptible to systemic deterioration due to the adverse effects of cancer treatments. Furthermore, HBOT does not contribute to cancer recurrence, making it a viable treatment option in patients with cancer. In the present case, no adverse events were observed during or after HBOT. Although HBOT is considered a useful technique with minimal adverse effects, there are reports of its ineffectiveness in cases such as the acute phase of electrochemical esophageal ulcers due to button battery ingestion,^10^ indicating that it may not be effective in all esophageal ulcers. Therefore, treatment selection should consider the patient's background and evaluation of treatment efficacy; consideration of alternative options is necessary if HBOT is ineffective. We suggest that EGD performed after 10 HBOT sessions might indicate efficacy.

In conclusion, we encountered a case wherein HBOT was effective in treating radiation‐induced ulcerative esophageal strictures resistant to conventional treatments. This report shows that HBOT has potential as a new non‐stricture‐resulting option in patients with ulcerative esophageal strictures. Further studies involving a larger number of patients across multiple facilities are warranted.

## CONFLICT OF INTEREST STATEMENT

None.

## ETHICS STATEMENT

All the procedures were performed in accordance with the ethical standards of the Declaration of Helsinki and its later amendments.

## PATIENT CONSENT STATEMENT

Written informed consent was obtained from the patient.

## CLINICAL TRIAL REGISTRATION

N/A
